# Uses of Energy Psychology Following Catastrophic Events

**DOI:** 10.3389/fpsyg.2022.856209

**Published:** 2022-04-25

**Authors:** David Feinstein

**Affiliations:** Independent Practice, Ashland, OR, United States

**Keywords:** acupressure, acupuncture, catastrophic events, disasters, emotional freedom techniques, energy psychology, thought field therapy

## Abstract

Energy psychology, as most widely practiced, integrates the manual stimulation of acupuncture points with imaginal exposure, cognitive restructuring, and other evidence-based psychotherapeutic procedures. Efficacy for energy psychology protocols has been established in more than 120 clinical trials, with meta-analyses showing strong effect sizes for PTSD, anxiety, and depression. The approach has been applied in the wake of natural and human-made disasters in more than 30 countries. Four tiers of energy psychology interventions following the establishment of safety, trust, and rapport are described, including (1) immediate relief/stabilization, (2) reducing limbic arousal to trauma-based triggers, (3) overcoming complex psychological difficulties, and (4) promoting optimal functioning. The first tier is most pertinent in psychological first aid immediately following a disaster, with the subsequent tiers progressively being introduced over time with complex stress reactions and chronic disorders. Advantages of adding the stimulation of acupuncture points to a conventional exposure approach are identified, and challenges around cultural sensitivities and unintended effects are discussed. After establishing a framework for introducing energy psychology in disaster relief efforts, reports from a sampling of settings are presented, based on interviews with this paper’s author. These include accounts of relief work with survivors of mass shootings, genocide, ethnic warfare, earthquakes, hurricanes, tornadoes, floods, wildfires, and the COVID-19 pandemic. Hundreds of other reports from the field show a pattern of strong outcomes following the use of energy psychology in the days or weeks after a disaster and in the subsequent treatment of trauma-based psychological problems. Many of these accounts corroborate one another in terms of rapid relief and long-term benefits. Finally, examples of more efficient delivery methods utilizing large groups, lay counselors, digital technology, and cultivating community resilience are presented.

While natural disasters capture headlines and national attention short-term, the work of recovery and rebuilding is long-term.

–Sylvia Mathews Burwell

Former U.S. Secretary of Health and Human Services

## Introduction

Energy psychology is a novel treatment for emotional healing and psychological development that involves the somatic stimulation of acupuncture points (acupoints) by tapping on them with the fingertips. Evidence for its speed and effectiveness has been rapidly accumulating, demonstrated in more than a 120 clinical trials and reviewed in the book, *The Science of Tapping* (Stapleton, 2019). Stapleton refers to energy psychology and other somatic interventions as comprising the “fourth wave” of psychotherapy, following psychoanalysis, behavior therapy, and cognitive approaches (p. xxiii). A credible estimate by Harvard psychiatrist Leskowitz (2016) placed the number of therapists incorporating techniques from energy psychology into their practices in the “tens of thousands” (p. 181). When brought to individuals and communities following a disaster, the performance of the approach has been particularly promising due to its ability to rapidly regulate the physiological aftermath of trauma, an assertion we will be examining in this paper.

## Incidence and Psychological Consequences of Major Disasters

The number of major recorded *natural* disasters in the decade from 2010 to 2019 worldwide nearly doubled compared with the decade of 2000 to 2009 (from 4,212 to 7,348) as “extreme weather events have come to dominate the disaster landscape in the 21st century” (United Nations Office for the Coordination of Humanitarian Affairs [OCHA], 2020, p. 1). Meanwhile, an analysis of databases focusing on *human-made* disasters in industrialized countries in the 20th century revealed “an exponential growth” in their frequency as well (Coleman, 2006, p. 3). Mass shootings, terrorism, genocide, warfare, and violent conflicts impacting civilian populations combine with an increased frequency of industrial accidents in producing this escalation.

Whether natural or human made, disasters lead to serious disturbances and disruptions not only to the lives of individuals but to “the functioning of a society” (Karácsonyi et al., 2021, p. 28). Disaster response management has focused on physical and economic needs, while the mental health issues caused by disasters have often been a “neglected area” (Makwana, 2019, p. 3091). Among the mental health issues enumerated by Makwana, in addition to Post Traumatic Stress Disorder (PTSD), are widespread anxiety, depression, shock, despair, grief, sadness, anger, denial, maladaptive behaviors, substance abuse, insecurity, sleep disturbances, moodswings, suspiciousness, paranoia, obsessions, loss of accustomed role in the community, and stress-related physical illness. These psychological effects of disaster have been found to be particularly severe among children, women, and dependent elderly populations.

## Psychological Interventions Following Disasters

Mental health service providers “first began responding to disasters in large numbers in the 1990s” (DeAngelis, 2014, p. 63). They originally used group interventions in which survivors, responders, and family members would all share their experiences. Interventions today are more refined. They involve recognition that, for most people, a natural recovery process will occur over time. Appropriate interventions in the days following a disaster include easing agitation, explaining and normalizing extreme emotional responses, promoting a sense of safety and stabilization, facilitating connections with support systems, and making referrals for those in need of more focused psychological support. Mental health crisis teams usually work in conjunction with disaster relief agencies such as the Red Cross as well as local community resources.

DeAngelis based her article, “What Every Psychologist Should Know About Disasters,” on interviews with pioneers in developing and delivering psychological interventions following catastrophic events, along with the special November 2011 issue of the *American Psychologist* on the theme of “9/11 10 Years Later.” While emphasizing that many people show resilience after a disaster without receiving psychotherapy, she also noted that for those who do show severe distress or difficulties functioning, psychological first aid can help them to restabilize and pull their lives back together. For those who are still traumatized several weeks after the disaster, various forms of crisis counseling have been developed.

Lasting from a single session to however many sessions are needed, DeAngeles explains that crisis counseling differs from other forms of counseling by being more direct and pragmatic, focusing on practical as well as emotional concerns. Crisis counseling also emphasizes skills for psychological recovery. Simple techniques for managing stress might include diaphragmatic breathing, journaling, or engaging in activities that are pleasurable and grounding. Cognitive techniques for reframing the experience and the conclusions drawn from it are also utilized in crisis counseling, as are exposure methods for reducing limbic system responses to triggering memories and cues.

One of the first organizations to systematically bring mind-body interventions into post-disaster deployments is the Center for Mind-Body Medicine in Washington, D.C. In a study of its approach conducted 5 years after the war in Kosovo had ended in 1999, 82 adolescents who had still been children during the war and who met the criteria for PTSD were randomly assigned to a 12-session group program learning mind-body techniques or a wait-list control group (Gordon et al., 2008). The program included “meditation, guided imagery, and breathing techniques; self-expression through words, drawings, and movement; autogenic training and biofeedback; and genograms” (p. 1469). Decreases in the PTSD symptoms of those who went through the program reached a high degree of statistical significance in comparison with the control group (*p* < 0.001). Benefits were maintained on 3-month follow-up. The control group then went through the program and showed similar improvements on the pre/post-measures. The Center has for some three decades been offering and expanding its mind-body programs for addressing ‘‘population-wide psychological trauma and stress^1^.”

## Comparison Studies of Psychological Interventions Following Catastrophic Events

Several studies have used meta-analytic methods to compare the outcomes of psychotherapies that have been applied following disasters or other forms of severe mental duress (Brown et al., 2017; Morina et al., 2017; Purgato et al., 2018; Bangpan et al., 2019; Mavranezouli et al., 2020a; van Ginneken et al., 2021). These reviews found a range in the effectiveness of the various methods studied for reducing trauma-based symptoms and improving functioning. The majority of approaches studied were variations of Cognitive Behavioral Therapy (CBT), such as Narrative Exposure Therapy (NET) and a trauma-focused form of CBT (TF-CBT). CBT is considered by many to be the “Gold Standard” for treating serious psychological conditions (David et al., 2018). However, other therapies were also included in the comparisons, such as meditation, play therapy with children, family therapy, Eye Movement Desensitization and Reprocessing (EMDR), Thought Field Therapy (TFT), and the Emotional Freedom Techniques (EFT).

Both TFT and EFT are forms of energy psychology, the focus of this review. In each of the six studies cited above, an energy psychology modality was included in the comparison, and the approach demonstrated strong outcomes. For instance, Brown et al. (2017) conducted a meta-analysis of psychological treatments for children who had experienced the effects of trauma following manmade or natural disasters. Only one of the 36 studies used an energy psychology approach, TFT. Effect sizes ranged from 0.09, a small effect, to 4.19, an extremely large effect. The average pre-treatment to post-treatment effect size across the groups was 1.47, a large effect. The largest effect of the treatments investigated, 4.19, was produced by TFT. In the comparison study by Mavranezouli et al. (2020a), EFT was among 17 interventions reviewed for treating traumatized youth. EFT was one of the two most effective therapies in reducing PTSD symptoms at treatment endpoint and the most effective of the 17 interventions in retaining improvement in PTSD symptoms on follow-up. Tapping therapies have, in fact, held up well to the “gold standard” of CBT. Of 10 head-to-head studies comparing the two modalities, all 10 found at least equivalent outcomes and, in several studies, the energy psychology protocols outperformed CBT in speed and durability on follow-up (reviewed in Feinstein, 2021b).

Eight of the ten head-to-head comparisons between CBT and energy psychology approaches were randomized controlled trials (RCTs). One of the two that was not a controlled trial gives a feel for the differences as experienced by those receiving the treatments. It was a retrospective study in Kurdistan with individuals who had experienced ongoing violence, atrocities, and political upheavals during and after the Iraq War (Seidi et al., 2021). Treatment outcomes were evaluated for clients who had been assigned to a single psychotherapist over a 2-year period. The therapist had been trained in CBT and subsequently in TFT. Thirty-one clients met the study criteria and were assigned to CBT or TFT using purposive sampling. Of the 13 clients who received traditional CBT treatments, one improved and the others showed either no change in symptoms, deterioration of symptoms, or dropped out of treatment. All 11 clients who received the TFT treatment showed symptomatic improvement. Seven of those who had received CBT treatment and showed no improvement and no promise of improvement (reasons given by the therapist included cultural factors, education level, difficulty applying theoretical concepts such as overgeneralization, failure to complete homework assignments, and fatigue from the number and length of therapy sessions) were subsequently provided TFT treatment. The TFT treatments led to improvement in each case.

While it is certainly possible that the single therapist in this study was simply more adept with TFT than CBT, the therapists I have interviewed for this and previous reports who learned an acupoint tapping protocol after having used CBT in their practices uniformly commented that adding tapping to conventional exposure techniques increased the speed and power of methods that involve revisiting traumatic events. Mollon (2008), for instance, has written that energy psychology is not an alternative to CBT, but a “crucial additional component that greatly enhances its efficacy,” providing more effective means for “affect regulation, desensitization, and pattern disruption” (p. 619). We will later explore the reasons that acupoint tapping combined with exposure techniques is proving to be more effective than exposure techniques alone.

## The Nature of Energy Psychology

Energy psychology is an umbrella term for treatment approaches that incorporate an “energetic” component (Feinstein, 2022b) into the psychotherapeutic process, often adapted from time-honored healing and spiritual systems such as yoga and qi gong (Gallo, 2004). The somatic stimulation of acupoints by tapping on them is the most widely used and well-investigated technique within energy psychology. More than 120 clinical trials demonstrate the efficacy of acupoint tapping as a psychotherapeutic intervention, often with unusual speed and durable outcomes (Feinstein, in press). A recent meta-analysis has shown that acupoint tapping is an essential ingredient for these strong effects (Church et al., 2020). The studies reviewed compared acupoint tapping protocols with otherwise identical protocols except that a different intervention—such as diaphragmatic breathing or tapping on “sham points”—was substituted for the acupoint tapping component. Most therapists who incorporate acupoint tapping into their practices do not identify it as their primary modality but rather integrate the technique into their existing clinical frameworks (Feinstein, 2016).

A mechanism by which acupoint stimulation enhances clinical outcomes involves the generation of signals that activate or deactivate specific brain regions. A 10-year research program at Harvard Medical School using imaging devices to investigate the effects of stimulating acupuncture points using traditional needling found that certain points send signals to the amygdala and other parts of the limbic system which reduce arousal almost instantly (Fang et al., 2009). Although acupuncture and energy psychology are vastly different practices, traditional needling on an acupoint and stimulating it manually have been shown to generate similar effects. For instance, a double-blind study comparing penetration by acupuncture needles with non-penetrating pressure that simulates the sensation of penetration found equivalent clinical improvements for both interventions (Takakura and Yajima, 2009).

The few imaging studies of acupoint tapping within a psychotherapeutic context to date have revealed brain changes that correspond with clinical improvement. For example, an fMRI study of acupoint tapping treatments with obese individuals showed that brain regions involved with food cravings that were activated when images of junk foods were shown before treatment were no longer activated after the treatment (Stapleton et al., 2019). This decreased brain activation paralleled a diminished desire for those foods. Other imaging studies have shown that acupoint tapping increased activity in frontal executive regions that are involved with rational choices and the management of emotional responses in stressful situations (Di Rienzo et al., 2019; König et al., 2019). This ability to activate or deactivate targeted regions of the brain by combining acupoint tapping with the mental activation of issues of concern is perhaps a cardinal advantage of the method. It is presumably at the core of the unusually rapid elimination of maladaptive stimulus-response pairings, as demonstrated in the clinical trials. Beneficial effects on the vagus nerve’s regulatory and social engagement functions have also been observed following acupoint tapping treatments (Schwarz, 2018).

Meta-analyses of acupoint tapping protocols applied in the treatment of anxiety, depression, and PTSD—three of the diagnostic categories that appear the most frequently in clinical practice—showed large effect sizes (above 0.8) for each condition. In the meta-analysis focusing on the treatment of anxiety, 14 RCTs included a total of 658 participants (Clond, 2016). The overall effect size for these 14 studies, pre-treatment to post-treatment, was 1.23. In the study of depression, 12 RCTs with a total of 398 participants had an overall pre-treatment to post-treatment effect size of 1.85 (Nelms and Castel, 2016). In the analysis of PTSD treatments, seven RCTs with a total of 247 participants also demonstrated an unusually high effect size (2.96; Sebastian and Nelms, 2017). Other psychological conditions that have been shown to respond to acupoint tapping, based on studies listed in a database maintained by the Association for Comprehensive Energy Psychology^2^, include phobias, anger, stress, concentration difficulties, food cravings, insomnia, and performance blocks. Physical conditions that have shown statistically significant improvement after acupoint tapping include fibromyalgia, pain, headaches, frozen shoulder, psoriasis, obesity, immune function, inflammation, and cardiovascular function.

While the meta-analytic reviews show strong effect sizes for using tapping protocols with PTSD and depression, the efficacy studies to date have not distinguished between single-incident PTSD and complex PTSD or between symptoms of depression and major depressive disorders (Feinstein, in press). Clinicians should, therefore, proceed cautiously with the more severe forms of either condition. Another caveat is that little data exists at this point on the effectiveness of tapping protocols in reversing conditions such as psychotic disorders, dementia, autism, bipolar, or deeply ingrained personality disorders. An occasional limitation due to the mechanics of the approach is that because tapping can appear “odd,” not everyone feels comfortable doing it. The safety of a clinical modality is of particular concern when working with disaster survivors. The risk of retraumatizing people while attempting to help them overcome emotional difficulties following catastrophic events is an ongoing challenge for trauma therapists (Duckworth and Follette, 2011). Acupoint tapping protocols seem less vulnerable to this risk than many other methods because they “are designed to approach distress in a graduated and tolerable way, titrating exposure to otherwise unbearable trauma that may have previously overwhelmed the client’s coping capacities” (Mollon, 2013, p. 355). A review of clinical trials of energy psychology treatments involving more than a thousand subjects found that no adverse events were reported (Church, 2013). Schulz (2009) conducted in-depth interviews with 12 psychologists working with adult survivors of childhood sexual abuse and reported that a common theme throughout the interviews was that energy psychology protocols were seen as allowing clients to “relieve the trauma in a non-invasive manner [that] lessens the possibility of retraumatization” (p. 17).

## A Different Type of Exposure

Psychological exposure involves the use of imagination or recall to mentally evoke an anxiety-provoking situation in a safe context. The procedure is applied to reduce the threat response to fear triggers, and it has been found to be “highly effective for patients with anxiety disorders, to the extent that exposure should be considered a first-line, evidence-based treatment for such patients” (Kaplan and Tolin, 2011, p. 33). Frequently used in working with PTSD, the most fundamental difference between acupoint tapping protocols and other psychological approaches to long-term emotional healing following disasters may be in the components of the procedure.

### Conventional Exposure Treatments

A 2008 study by the Institute of Medicine (IOM) of the National Academy of Sciences, *Treatment of Posttraumatic Stress Disorder: An Assessment of the Evidence*, found that despite nearly three decades of research since the adoption of PTSD as a formal diagnostic category, the existing studies “do not form a cohesive body of evidence about what works and what does not” (Committee on Treatment of Posttraumatic Stress Disorder, 2008, p. 10). The single type of intervention (psychological or pharmaceutical) whose efficacy was judged as having been empirically established, however, was prolonged imaginal exposure.

While the view that exposure is necessary for the successful treatment of PTSD has been questioned, in part due to the possibility of retraumatization (Farrell et al., 2013), and alternative treatments have been proposed (Markowitz et al., 2015), prolonged exposure is still the most widely recommended approach for treating PTSD. The American Psychological Association’s (2017) *Clinical Practice Guideline for the Treatment of Posttraumatic Stress Disorder* strongly endorsed prolonged exposure therapy, or prolonged exposure therapy with cognitive restructuring, as the primary recommended interventions for treating PTSD. Eye Movement Desensitization and Reprocessing (EMDR) was given a conditional recommendation. Energy psychology was not evaluated.

### Inconsistencies in Clinical Experiences With Exposure Treatments

Although EMDR and acupoint tapping are very different approaches, both utilize imaginal exposure and combine it with a somatic intervention. The somatic interventions in EMDR may include bilateral stimulation in the form of back-and-forth eye movements, alternating buzzers, or other means of mixing right-left stimulation, such as tapping, alternately, on the knees, legs, or shoulders. As efficacy studies were establishing EMDR as an effective treatment for PTSD, outcome data began to accumulate that were not consistent with the guidance derived from experiences with other exposure treatments (Rogers and Silver, 2002). Rogers and Silver noted, for instance, that “previous research suggests that repeated brief exposures only result in fear decrement when stimulus intensity and arousal are both low. Yet EMDR uses very brief (20-30-s) exposures [even though] stimulus intensities are high, since clients are asked to start by focusing on the most distressing scene” (p. 49).

Four differences between *conventional formulations of exposure therapy* (as delineated in the literature establishing the approach) and *exposure paired with a somatic intervention* (based on reports from clinicians) have been delineated (Feinstein, 2010) and are summarized in Table 1.

**TABLE 1:** Two contrasting approaches to exposure.

Conventional Exposure Treatments	Exposure with a Somatic Intervention
(1) Brief exposure, as is used in systematic desensitization (10 to 15 s in each round of the protocol), may be effective for low levels of arousal, but not for highly distressing stimuli. In addition, a large number of sessions over an extended period of time is required for brief exposure to be effective even with low levels of arousal (Rothbaum and Foa, 1996/2007).	(1) Brief exposure combined with acupoint stimulation has been found to be effective with conditions that involve high as well as low levels of arousal, and a few rounds of brief exposure during a single therapy session are often able to uncouple the association between a stimulus and a maladaptive fear response.

(2) Prolonged exposure is in fact generally needed in the treatment of anxiety disorders, with 20 min often being required before the anxiety associated with a simple phobia begins to diminish and up to 60 min with agoraphobia (Foa et al., 1989). For trauma scenes, up to 100 min of flooding (where anxiety-provoking triggers are presented in an intense, sustained form) were required before decreases in anxiety were reported (Keane, 1995).	(2) Prolonged exposure or a long series of repeated exposures are not required to obtain desired clinical outcomes.

(3) Clients are required to “focus their attention on the traumatic material and… not distract themselves with other thoughts or activities” (Brewin, 2005, p. 272). In fact, allowing the client to shift away “from the most traumatic cues” is believed to be “no more effective in attaining extinction to the anxiety than past episodes of intrusive recall have been” (Lyons and Keane, 1989, p. 147).	(3) The focus during the exposure sessions is not fixed but is instead, while the tapping continues, allowed to shift among traumatic memories and other thoughts, beliefs, physical sensations, emotions, and expectations.

(4) Exposure works for fear and anxiety but does not seem effective in the treatment of guilt or other complex emotions that require higher order cognitive constructs (Foa and McNally, 1996).	(4) Emotions that require higher-order cognitive constructs such as guilt, shame, or grief have responded to the approach.
	

*Sources for the “Conventional Exposure Treatments” boxes include observations from developers of the approach which still correspond with current practices. The “Somatic Component” boxes were corroborated in an analysis of over 800 interviews or survey responses from energy psychology practitioners (Feinstein, 2021a).*

### Conventional Exposure Methods Do Not Eliminate the Original Fear Learning

What might account for these differences? A surprising discovery within neuroscience was that conventional exposure approaches *overwrite* rather than *replace* the learnings that generate fear while other approaches are able to generate a new learning that completely *eliminates* the old learning, a difference with substantial clinical implications (Dunsmoor et al., 2015). Specifically, if the old associations are overwritten instead of eliminated, the client is vulnerable to recurrences via (a) *spontaneous recovery* of the conditioned fear response, (b) *renewal* of the fear when the original cue is presented outside of the extinction context, or (c) *reinstatement*, where the original aversive stimulus is presented without the original cue but renews the original cue’s ability to trigger a fear response. If the old associations have been neurologically extinguished, however, these recurrences would not be possible without the occurrence of a new traumatic event.

Frequent recurrences of the old learning following conventional exposure treatments have been found in both laboratory and clinical settings (Dunsmoor et al., 2015). Exposure was not proving to be as effective as hoped, even with the addition of cognitive restructuring techniques. In fact, recent studies and meta-analyses have suggested that CBT, with exposure treatments being one of its primary strategies, only slightly outperforms the placebo effect (Leichsenring et al., 2018), a controversial but provocative finding.

The initial commonsense understanding of exposure therapy had been that extinction (the elimination of a conditioned response such as a fear of spiders) is brought about by the eradication of an old association through repeated presentations of the trigger (the conditioned stimulus) in a safe context. But because the response could spontaneously return, it was undeniable that the original learning had not been eliminated, requiring that the theory be revised. Foa and McNally (1996) explained that based on abundant evidence, “fear reduction does not involve the weakening of associations *per se*, but rather involves the formation of new associations [that] override the influence of pathological ones” (p. 339).

Meanwhile, neurochemical studies indicated that the administration of certain drugs when paired with fearful experiences make it possible to eradicate old fear associations at the neurological level (Berlau and McGaugh, 2006). This was deduced by multiple experiments showing that the original fear couldn’t be reactivated using any known methods for reactivating extinguished fears. Another, more powerful mechanism than the accepted understanding of extinction had been discovered. It explained a second way the brain updates itself on the basis of new experience. This was further demonstrated when behavioral rather than pharmaceutical interventions were created that completely removed the fear, first with laboratory animals (Monfils et al., 2009) and then with human subjects (Schiller et al., 2010). Monfils et al. noted that comparisons of conventional extinction methods with those that eliminate rather than override old associations “engage different mechanisms in the lateral amygdala and lead to a drastically different behavior outcome” (p. 953).

Another prevailing belief among neuroscientists had been that once a new learning is consolidated into long-term memory, it is permanently installed. It could be modified, or even eclipsed by subsequent experiences, as in the extinction process brought about by conventional exposure techniques, but it nonetheless remained and could be reactivated. Hundreds of studies over several decades have shown, however, that this is not the only possibility. Rather, “a consolidated memory can…be modified, strengthened, changed or even erased!” (Nader, 2003, p. 65). For the learning to be erased, a sequence must occur in which the outcome predicted by the original learning does not take place, a mismatch neuroscientists call a “prediction error” (Exton-McGuinness et al., 2015).

### Combining Acupoint Tapping With Psychological Exposure

Acupoint tapping protocols accomplish this by having the client mentally activate the old learning—we’ll stay with the fear of spiders—while sending deactivating signals to the amygdala via acupoint tapping. After a few rounds of tapping, the image of a spider can be vividly accessed but the expected fear is not experienced. This process is illustrated in a 13-min video showing excerpts from a 30-min treatment (available at http://phobiacase.EnergyPsychEd.com, accessed February 10, 2022). As you can see by the surprise on the client’s face, a prediction error has been created. This is the basic sequence necessary for the neural pathways that maintain the old learning to become “depotentiated” at the synaptic level. The learning is reconsolidated in a new way, one that corresponds with the recent experience. A more detailed explanation of this process is available elsewhere (e.g., Feinstein, 2019), but for the purposes of understanding the role of energy psychology in disaster relief work, the key concepts are that: (a) *brief* exposure is adequate if accompanied by acupoint tapping, (b) retraumatization can be prevented, and (c) the changes are lasting.

## Energy Psychology in the Treatment of Disaster Survivors

Energy psychology has been applied in the wake of natural or human-made disasters in Australia, Bosnia, Brazil, Burundi, Columbia, the Democratic Republic of Congo, Ecuador, Germany, Guatemala, Haiti, India, Indonesia, Israel, Japan, Kenya, Kosovo, Kuwait, Liberia, Mexico, Moldavia, Nairobi, New Zealand, Nicaragua, Nigeria, Rwanda, Sierra Leone, South Africa, South Sudan, Sweden, Tanzania, Thailand, Venezuela, Uganda, and the United States. Teams in the United States have worked with survivors of fires, earthquakes, hurricanes, tornadoes, industrial accidents, and school shootings as well as communities and health care institutions hard-hit by the COVID-19 pandemic.

Several international humanitarian relief organizations have adapted energy psychology as a treatment in their post-disaster missions. Capacitar International (‘‘Capacitar’’ is a Spanish word meaning to empower, to awaken, to bring each other to life) has been working with communities in transition -- including those in the aftermath of trauma, violence, war, and other disasters -- since the 1980s^3^. Their staff has won multiple international awards, and their reach has extended into five continents. Among Capacitar’s primary somatic interventions are EFT, TFT, and other acupressure techniques for alleviating physical and emotional pain. Charles Figley, who served as the chair of the committee of the Department of Veteran Affairs that first named PTSD and who is also the founder of Green Cross, allowed this to be posted on an energy psychology website in 2005: “Energy psychology is rapidly proving itself to be among the most powerful psychological interventions available to disaster relief workers for helping the survivors as well as the workers themselves.”

Most individuals who bring psychological services to a disaster area follow the generally accepted guidelines articulated by DeAngelis (2014) in terms of prioritizing the alleviation of distress, explaining and normalizing extreme emotional reactions, facilitating stabilization and a sense of safety, linking survivors with appropriate support systems, and collaborating with the local community and any relief agencies that have been deployed.

### An Early Study

**FIGURE 1:**
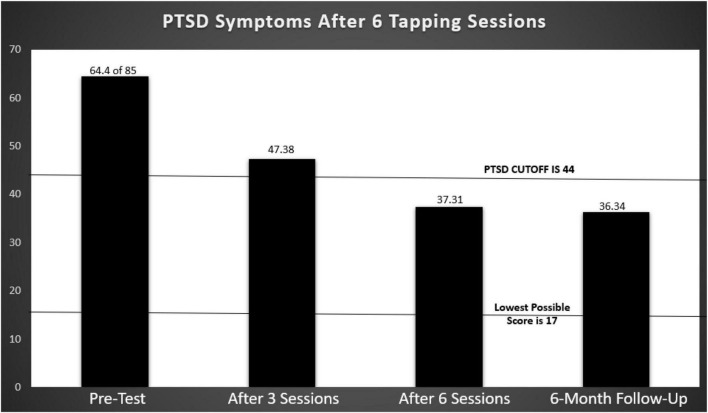
Post Traumatic Stress Disorder Checklist-Military (PCL-M) scores before and after PTSD tapping treatments with 49 combat veterans. Based on Church et al. (2013).

The first RCT investigating the use of energy psychology with PTSD involved 49 combat veterans who scored in the PTSD range on a symptom checklist that had been standardized with veterans (Church et al., 2013). Dramatic improvement was found after six treatment sessions, with 42 of the 49 participants no longer scoring above the PTSD cutoff. The participants had been recruited from throughout the United States and treated by volunteer practitioners. The gains persisted at 6-month follow-up (see Figure 1). The study has been replicated with similar findings (Geronilla et al., 2016).

By way of comparison, approximately two-thirds of service members and veterans completing a course of prolonged exposure treatments and/or cognitive processing therapy in peer-reviewed studies published between 1980 and 2015 still met PTSD diagnostic criteria after treatment (Steenkamp et al., 2015). There was only one drop-out in the Church et al. study. In contrast, nine of every ten of the 49,425 veterans of the Iraq and Afghan wars with newly diagnosed PTSD who sought care from facilities run by the U.S. Department of Veterans Affairs dropped out before completing the conventional treatments as recommended (Seal et al., 2010).

### Four Tiers of Energy Psychology Interventions Following Catastrophic Events

While exposure treatments are generally used for long-term healing rather than immediately following a disaster, acupoint tapping protocols can be applied at any point after a catastrophic event. If a person is experiencing acute trauma, it is not necessary for them to imagine the traumatic event. They are already in it. You can direct them to tap to immediately decrease their emotional distress, as with any other potent relaxation technique. Tapping can then also be applied at a later stage of recovery for defusing intrusive or otherwise unprocessed memories about the trauma. After attending to physical needs, establishing safety, and fostering trust and rapport, a four-tier framework categorizes energy psychology interventions in post-disaster situations according to their purpose (Feinstein, 2008):

#### First Tier: Immediate Relief/Stabilization

Much as a paramedic might instruct a patient having an anxiety attack in a breath control technique that is incompatible with hyperventilation, energy psychology utilizes interventions which rapidly down-regulate the limbic system mediated fight-or-flight response. Tapping on specified acupuncture points whose stimulation has been shown to decrease activation signals in the amygdala (Hui et al., 2005), for instance, appears to rapidly decrease elevated emotional responses in stressful situations. This simple procedure can be a potent intervention for providing psychological first aid in the immediate aftermath of disaster. Practitioners often start with the most comforting interventions available for fostering relief and stabilization–such as diaphragmatic breathing, self-hugs, gentle rocking, and offering reminders that the person survived and is safe now—introducing tapping as appropriate.

#### Second Tier: Reducing Limbic Arousal to Trauma-Based Triggers

Beyond immediate relief, acupoint tapping can be applied to change maladaptive stress response patterns that develop after a disaster. Amplified fear, rage, or anguish may have become neurologically associated with a particular internal or external cue. By reducing limbic hyperarousal in the presence of the cue, self-defeating affective, cognitive, and behavioral patterns may be interrupted, including avoidance behaviors, a learned proclivity that serves to reinforce PTSD (Badour et al., 2012). Uncoupling extreme stress responses from memories, chilling fantasies, or external triggers is a key to the successful treatment of PTSD (van der Kolk, 2014).

#### Third Tier: Overcoming Complex Psychological Difficulties

Complex issues involving early attachment experiences, current relationships, personal goals, coping styles, work setting, and physical health may have surfaced because of the trauma experience and can be effectively addressed, particularly once adequate progress in the first two tiers has been accomplished. An energy psychology approach is able to identify and target salient aspects of complex problems. Contributing factors to low self-esteem, for instance, might include unresolved memories of parental emotional abuse, self-defeating beliefs, exaggerated appraisals of interpersonal threat, and anxiety in social situations. The combination of acupoint stimulation with the mental activation of carefully selected scenes, feelings, or beliefs may be applied to the elements of a complex psychological problem, one by one. A tapping protocol for “neutralizing negative core beliefs and for instilling positive ones” (Gallo, 2004, p. 181) is often applied. Whether using tapping to resolve an earlier traumatic memory that is tied to obstacles in overcoming the more recent trauma or addressing a childhood belief that contributes to pessimism and hopelessness, untangling such constellations frequently becomes a focus of ongoing treatment and may be necessary in post-disaster counseling for a complete healing to occur.

#### Fourth Tier: Promoting Optimal Functioning

Even after having restored stabilization, neutralized limbic responses to traumatic cues, and taken steps toward resolving self-defeating patterns that trace to childhood, the existential issues of life remain. In fact, a catastrophic experience may accentuate questions of meaning, uncertainty about the future, the reality of evil, and awareness of the inevitability of death. Yet people who have seen the worst of life do prevail emotionally and spiritually. As Tolstoy observed, “There is something in the human spirit that will survive and prevail, there is a tiny and brilliant light burning in the heart of man that will not go out no matter how dark the world becomes.”

Many people, in fact, discover that previously unknown strengths and resilience follow a catastrophic event. The term *post-traumatic growth* describes “positive psychological changes that are experienced as a result of struggles with highly challenging life circumstances” (Jayawickreme and Blackie, 2014, p. 312). Interviews with energy psychology practitioners suggest that an energy-attuned approach can help uncover and strengthen that “tiny and brilliant light,” fostering feelings of spiritual connectedness and promoting serenity, confidence, and courage (Feinstein, 2021a). Although these are ongoing issues and often involve intense challenges, greater personal stability and a higher level of functioning are attainable outcomes following traumatic experiences.

At these third and fourth tiers, energy psychology is often integrated with other clinical or personal development approaches. In enhancing personal resilience, for instance, strategies from Positive Psychology (such as the “building of buffering strengths” like perseverance or a capacity for pleasure, Seligman, 2002, pp. 6-7) may provide a framework as energy psychology techniques are employed to instill such strengths.

### Situational Considerations

Beyond the method is the context. Energy psychology interventions in post-disaster settings must be applied with an understanding of the phases of disaster relief, a sensitivity to cultural issues, vigilance about counter-intuitive dynamics that often accompany psychological interventions following catastrophic events, and the practitioner’s own vulnerabilities. Each is discussed in this section.

#### Calibrating Interventions to the Three Phases of Disaster Relief

Applications of energy psychology following a disaster need to be calibrated to the unique needs and constraints of each individual and to an understanding of the kinds of intervention that are appropriate at various timeframes after a disaster. A seminal volume, *Interventions Following Mass Violence and Disasters: Strategies for Mental Health Practice* (Ritchie et al., 2007) includes chapters discussing principles for *immediate responses* to disaster (Ruzek, 2007; Young, 2007; Ørner et al., 2007), interventions *1 to 4 weeks* after exposure to a trauma (Bryant and Litz, 2007), and longer-term interventions (Raphael and Wooding, 2007). The four tiers of energy psychology interventions are keyed to these three phases of disaster relief interventions in Feinstein (2022a).

#### Cultural Sensitivity

In providing mental health interventions with disaster survivors, cultural and demographic considerations are sometimes critical (Norris and Alegria, 2007). While little empirical evidence exists based solely on work with disaster survivors to guide energy psychology practitioners in establishing differential treatments for specific stricken populations, MacKay and Alfred (2013) have outlined general principles for using tapping protocols with ethnic and cultural groups different from those of the practitioner. These can be applied in post-disaster situations as well.

Dr. Carl Johnson, a pioneer in applying energy psychology (specifically TFT) in post-disaster settings, emphasized in our interviews that treatment success can sometimes “hang on the use of a culturally or personally sensitive word.” An ABPP level clinical psychologist, Dr. Johnson retired from a career as a PTSD specialist with the Veterans Administration. He learned TFT toward the end of his time at the V.A. and found it to be far more effective than the tools that had previously been at his disposal. At the time of the interviews I conducted with him in 2005, Johnson had for the nearly two decades following his retirement regularly traveled to the sites of some of the world’s most terrible atrocities and disasters to volunteer psychological support using TFT.

He offered an example to illustrate the ways in which constraints on men about expressing emotional distress may make it challenging in post-disaster work to even name the specific issues that need to be mentally activated during the tapping. An ethnic Albanian who spoke English brought a former Kosovo Liberation Army soldier to Johnson’s hotel. The translator said, “He’s here for help with his war trauma.” Johnson explained the 0-to-10 scale and asked the man to give him a number for the intensity of his trauma. The translator conferred with the man and then said, “No number, none.” Johnson asked, “Isn’t he here because he is suffering from trauma?” The translator restated, “No number, no trauma.” Johnson continued:

I sensed that while the man had come for help, he was also obeying the Albanian taboo which forbids suffering in males. I decided to bypass any mention of his suffering and said to the translator, “Okay, but could you ask him to just think about the traumatic event.” The response: “No traumatic event.” It dawned on me that by definition, to qualify as a traumatic event, it would have had to cause a personal trauma, which he couldn’t admit to. So I asked if he had had a challenging experience, a bad moment that he had overcome.” To this, he could say “Yes.” So I had him think about the bad moment he had overcome. I asked him if he would enjoy having a tune-up on his strong body to get it ready for his next victory, like tuning up the engine of a magnificent race car that has won but needs to have a tune-up to win again.” He said, “That would be fine.” As he focused on the event he had overcome, I used an energy assessment procedure to find and then treat his energy disruptions. Finally when I could find no further disruptions in his energy system, I asked him if anything more had to be done or if the tune-up had been complete. He looked relaxed. Then he spoke through the translator: “He wants me to tell you he thanks you very much for healing his trauma.” Once the trauma had been resolved, it was no longer an issue for him to use the word.

Many variations of this story may be encountered by relief teams deployed to unfamiliar cultures. The use of energy psychology with children also requires calibration. Children respond at least as well as adults to tapping for reducing arousal, according to practitioner reports, but the approach must be framed at a level that is appropriate to the child’s age, situation, and level of understanding.

Even explaining energy psychology in terms that are respectful of and congruent with the person’s worldview and assumptions about healing may be problematic. Explaining an approach that is rooted in a paradigm adopted from ancient healing traditions has, in fact, proven to be a substantial challenge for Western energy psychology practitioners within their own culture. On the other hand, people from cultures rooted in an energy or spirit-based outlook, such as Native Americans and members of various African tribes, can often intuitively relate to energy psychology theories and methods.

#### Counter-Intuitive Snares

Common-sense assumptions about working with disaster survivors have sometimes been refuted by clinical observation, and various counterintuitive aspects of early post-disaster interventions have been identified. Ruzek (2007) discusses several assumptions at the core of various intervention models that should be examined rather than uncritically accepted. For instance, Critical Incident Stress Debriefing—in which trauma survivors share, within a supportive professional context, their experiences, thoughts, and emotional reactions with other survivors who were involved in the same trauma—once seemed to make a great deal of sense and was widely applied. Yet strong evidence shows that it can interfere with natural coping strategies in resilient people and increase rather than prevent PTSD incidence in vulnerable individuals (McNally et al., 2003).

Disaster relief workers are routinely taught to “normalize” acute stress reactions. This validates the natural resilience of survivors and helps them understand that their responses are normal and transient rather than signs of personal weakness or mental illness. It serves individuals for whom acute distress symptoms are going to be temporary, and it may be therapeutic since many affected individuals are highly suggestive immediately following a trauma. But it may also create negative consequences for survivors whose symptoms persist. Research on survivors of mass violence, in fact, shows high percentages with enduring problems, considerably higher than in survivors of *natural* disasters (Quarantelli, 2000). Overemphasis on the fact that most symptoms of acute stress reactions following trauma will spontaneously dissipate over time may, therefore, unintentionally stigmatize people who need treatment and ultimately keep them from receiving it. In short, people may, following a disaster, become extremely sensitive, their stability erratic, and their responses to seemingly innocuous statements surprising.

Another assumption, one that traces back to combat psychiatry, is that it is important for mental health specialists to actively intervene as soon as possible after a trauma. Various outcome studies, however, along with concerns about pathologizing normal reactions, give “reason to question whether intervening sooner will result in better care” (Ruzek, 2007, p. 20). Responders are taught that the most viable working assumptions 24 h after a disaster may be substantially different from the most viable working assumptions 3 weeks later or 6 months later. Applications of energy psychology following a disaster must be calibrated to the unique needs and constraints of each individual and to an understanding of the kinds of intervention that are appropriate at various timeframes after the disaster.

Other counter-intuitive dynamics have also been identified. For instance, Levine (1997) has shown that people (as well as animals) who shake and quiver after a trauma are less likely to develop PTSD symptoms. So holding and invasively soothing a person who is shaking may actually interfere with recovery.

Even meditation may lead to unintended outcomes (Lyford, 2022). Fay (2021) explains that meditation practices “can engender a state of bliss that may be scary for people with trauma histories. Saying anything even remotely positive can generate intense self-hatred…. In a blissful state, boundaries may be experienced as too diffuse, leaving us feeling unsafe or out of control. It can also trigger regression” (p. 39).

Given the widespread favorable reports in the clinical literature on the benefits of meditation, however, I was curious about its use in post-disaster situations. When doing the interviews for the “Reports from the Field” section below, I asked Dr. Lori Leyden if she had used meditation in working with the survivors of the Sandy Hook Elementary School shooting. She replied:

In my experience, meditation without the kind of somatic release that occurs with acupoint tapping is often contra-indicated for acute trauma and PTSD because the client’s physiology isn’t regulated enough to deal with intrusive memories and other trauma symptoms that may arise. This can leave them feeling even more unsafe in their bodies. I have witnessed a number of people, including parents of murdered children, being told by meditation teachers that meditation is an appropriate trauma healing tool even after the traumatized parents had expressed major discomfort while trying to meditate.

#### Disaster Relief Worker Vulnerability

A delicate issue is the vulnerability of the practitioner administering disaster relief services. The psychological health and stability of the healer is generally assumed to surpass that of the client, yet local health care providers have often been part of the same traumatic event as those they are serving. They may also be offering care to people whose stories are recent, horrific, and triggering. Providing training and support for working through their own unresolved traumatic experiences is a vital element in preparing health care personnel to provide disaster relief services. Because tapping therapies can yield rapid results and be self-applied, they can serve the practitioner’s personal healing and evolution while the practitioner is learning how to use them in post-disaster situations.

## Reports From the Field

In the decade following the 2001 attacks on the Pentagon and the World Trade Center, advances have been made in: (a) assessing the needs of individuals and communities impacted by catastrophic events, (b) creating programs that address those needs, and (c) evaluating those programs (Watson et al., 2011). Nonetheless, the unexpected nature, chaos, and pressing demands that suddenly emerge when a disaster strikes mitigate against systematic evaluation when trauma response teams arrive immediately after a catastrophic event.

The research on tapping protocols in post-disaster situations that does exist was usually conducted long after the disaster occurred, when systematic research procedures were more achievable. Positive outcomes after treating chronic PTSD in civilian populations in the years following a disaster have, in fact, been striking. For instance, in a clinical trial nearly two decades after the 1992-95 wars in Bosnia, 18 adults were selected based on severe ongoing emotional distress tracing back to their experiences during the wars, which included severe injuries, torture, beatings, rape, sexual humiliation, and watching others being assaulted or murdered. Each of the 18 participants received four 1-h sessions using a tapping protocol over a 2-week period. A standardized civilian PTSD symptom checklist was administered prior to the treatment, at the end of treatment, and at 4-week follow-up (Boath et al., 2014). Significant reductions were found in symptoms (*p* = 0.009) and sustained on follow-up.

In another study of civilian survivors of systemic violence years earlier, Connolly and Sakai (2011) randomly assigned 145 adult survivors of the 1994 genocide in Rwanda to TFT treatment or a waitlist control. Differences between the post-treatment and waitlist groups on PTSD symptom scales were highly significant (*p* < 0.001), with moderate to large effect sizes on changes in both the frequency and severity of symptoms. Imrpovements were sustained at 2-year follow-up.

While systematic research on energy psychology interventions immediately after a disaster is still unavailable, hundreds of reports from the field show a pattern of strong outcomes following the use of energy psychology in the days or weeks after a disaster and in the subsequent treatment of trauma-generated symptoms. Many of these accounts corroborate one another in terms of rapid relief and long-term benefits, yet the state of the art in applying energy psychology immediately following disasters still resides largely with the practitioners who have been carrying out such work. In the remainder of this section, I will present a small sampling of the accounts from those I interviewed (in person, telephone, Zoom, or e-mail) who have been bringing energy psychology to communities following natural or human-made disasters.

### Sandy Hook Elementary School Shooting

This widely reported heart-breaking tragedy occurred on December 14, 2012, in Newtown, Connecticut A 20-year-old former student of the elementary school shot and killed 28 people, including 20 children between 6 and 7 years old, six adult staff members, the shooter himself, and his mother. Nick Ortner, a long-time resident of Newtown, happens to have founded one of the most influential organizations promoting an acupoint tapping approach to healing and personal development^4^. His mother, Dr. Maria Ortner, was a school psychologist at a nearby elementary school. She had worked closely in the past with both the school psychologist and the principal who were killed at Sandy Hook.

Deeply touched on many levels, Nick was determined to bring his knowledge of energy psychology and EFT, as well as his local and global connections, to do something that would generate genuine healing for the traumatized community. On the day after the shootings, he contacted Dr. Lori Leyden, a colleague and internationally known trauma expert. She has introduced acupoint tapping and other disaster relief methods following some of the world’s worst recent disasters involving genocide and mass shootings^5^. Nick asked Dr. Leyden’s advice about providing an immediate response and facilitating long-lasting healing. That discussion turned into a collaboration. Three days later, Dr. Leyden arrived to begin organizing a long-term therapeutic and self-care initiative for the many people in Newtown who were affected by the shooting.

From the day Dr. Leyden arrived, she began conducting sessions with individuals as well as groups. Because the effects of long-term trauma are well-known, and because of her success working with other survivors of horrific violence, Dr. Leyden was able to immediately bring this experience to the task of establishing a community approach for Newtown that incorporates long-term, sustainable practices for relief. She made a commitment to the project and wound up living in Newtown for the next 3 years, though the work is ongoing nearly a decade later. The goal was “to come in quietly, listening and observing, supporting local efforts, and providing the team an unobtrusive method for assessing needs while also offering therapeutic and self-care assistance to those who needed it most.”

Nick sent out a request for volunteers to his 500,000 strong mailing list. Nick and Dr. Leyden then hand-picked 35 volunteer tapping practitioners, out of hundreds of responses, to help create and build a long-term model for Newtown. Training in applying their facility with acupoint tapping to post-disaster situations began on January 5, 2013, 22 days after the shooting. The volunteers spent 35 to 60 h in training, along with many more in supervision, to prepare for the immediate and long-term needs of those directly and indirectly affected by the tragedy. Particular focus and outreach went to the parents and other family members of those killed, the children who survived the shooting, the school’s teachers and other staff, and first responders, including police, firefighters, emergency medical technicians, medical examiners, and funeral directors. Rather than attempt to summarize the vast number of individual and group tapping sessions or related workshops and community events, a few comments by recipients or providers of the services follow (source: Ortner et al., n.d.):

Scarlett Lewis, Mother of 6-Year Old Jesse Lewis, Slain During the Shooting: “In my attempt to heal from the tragedy of losing my son, an experience that has broken my heart and made me question going on with my own life, I sought many different types of help. Initially I sought traditional ‘talk’ therapy that left me retraumatized and feeling worse. Nick Ortner introduced me to Tapping, and I always finish these sessions with a deeper understanding of myself, feeling better, with a lightness of being, and hope. Tapping makes me feel better when nothing else does…”
Physician and First Responder, from the Office of the Medical Examiner: “Dr. Leyden offered her services just days after the tragedy. She has been out to our office three times and done multiple sessions each visit, spending several hours with technicians, doctors, investigators, and other staff directly involved with the Sandy Hook shootings. Her tapping and breathing exercises, as well as the group discussions have been very helpful to me and my staff. She has continued to check in with me regarding our progress and offers to help us in any way she can. I personally am sleeping better and functioning better.”
Lynn Johnson, MS LPC LADC, Director, Center for Serenity, Hartford, CT. “I have been so honored and moved to be a part of this project. Dr. Lori Leyden, Nick Ortner, Jondi Whitis, and the whole group have really inspired me. I have developed a program for young children, called the “Feel Free Tap,” which is a version of EFT for grades K to 3. I loved sharing it with the group and can’t wait to take it out to the wider community!”
Bonnie Skane, Volunteer. “Being part of this volunteer team is such an honor and a blessing. In spite of this terrible tragedy, we have been seeing many little miracles happening every day. It’s such a joy to help someone who is experiencing tremendous emotional pain, anxiety, and stress find relief with EFT! I truly believe that we change the world by changing ourselves, and EFT is simply an amazing tool that gives us the ability to release our negative emotions and choose positive ones instead.
Alison Held, Volunteer: “There is significant positive change happening in Newtown and beyond as a result of tapping! The EFT Stress and Trauma Relief Project is unfolding in the most beautiful way imaginable, with a core community of talented volunteers with a clear and unified vision of hope, love, and healing.
Eric Leskowitz, a Psychiatrist at Harvard Medical School: Dr. Leskowitz provided this advice to the organizers: “Based on my clinical experience and reading of the research literature, EFT is the treatment of choice for rapid intervention in traumatic situations like Newtown that trigger overwhelming emotions in individuals and groups. Its use can prevent the future development of full-blown PTSD by empowering people to develop control over their own nervous systems.”

A poignant “full-circle” story following the Sandy Hook tragedy involved a 12-year-old boy whose 6-year-old brother was killed during the shooting. While the boy’s mother had quickly embraced tapping, the boy was highly skeptical. He was understandably extremely angry about losing his brother and hadn’t attended school since the tragedy 2 months earlier. Dr. Leyden had previously worked with orphaned genocide survivors in Rwanda, first for healing, but then teaching them to become “heart-centered” leaders. The program was later formalized as “Project LIGHT: Rwanda.” Graduates of the program are referred to as “Ambassadors,” and a goal of the initiative is to connect traumatized young people around the world to support one another.

A Skype meeting was arranged between the 12-year-old boy in Newtown and two of the Rwanda Ambassadors, young people like himself who had been through the worst of human tragedies. During the long call, they shared deeply, tapped together, and genuinely bonded. The boy in Newtown was so inspired that he returned to school the next day to make a speech to his classmates about why it is important to care about people who have experienced even worse tragedies. Completing the full circle, he went on to create a non-profit organization that raised money for two of the Rwanda Ambassadors to attend university. Several years later, he traveled to Rwanda for an emotional reunion with the Ambassadors who had helped him so much while he was deep in grief about his brother’s death.

### Refugee Camp Challenges

By the end of 2020, 82.4 million people worldwide had been forcibly displaced as a result of persecution, conflict, violence, human rights violations, or other events that seriously threatened their security (UN Refugee Agency, 2021). According to credible estimates, about one in three of these individuals suffer with chronic depression, anxiety, or PTSD (Turrini et al., 2017), and all are facing substantial mental health challenges.

The Moria Refugee Camp in Greece was Europe’s largest camp when Gunilla Hamne and Ulf Sandström, co-founders of the Peaceful Heart Network^6^, were called to the camp to assist with a disturbed 8-year-old boy who was “out of control.” The Peaceful Heart Network has developed an acupoint tapping approach, called the Trauma Tapping Technique (TTT), which relies on a minimal use of words. Derived from TFT and EFT, this streamlined approach is particularly well-suited for healing the wounds of trauma. It can readily be taught to disaster survivors and paraprofessionals or brought to large groups. In addition to tapping, TTT uses a self-soothing technique called “Havening,” another approach that employs touch to trigger electrochemical reactions in the body that can reduce escalated emotional responses to a memory or trigger (Ruden, 2019).

The boy in the Moria camp was acting out violently within his family and against others in the camp: “biting, throwing objects and stones, destroying tents, peeing everywhere, and tearing his clothes.” The father was very caring and patient and tried his best to manage the boy. The mother had become numb and passive. The whole situation was creating chaos in the family, and with people so cramped, it had reached the point where the family was at risk of being forced to leave the camp with no place else to go. Hamne and Sandström describe their experience:

We went to one of the tents and did some drawing and acrobatic activities to connect. Suddenly the boy started to destroy everything in the tent including the books and toys, fetching big stones and throwing them at everyone. He broke the metal legs of a table and demonstrated that he could use them as weapons. He demanded more pens, which he then broke into pieces. When the boy had calmed down, we demonstrated our exercises and techniques to the father and the other children of the family. The interpreter also participated. We had seen that the father could hold the boy and hug him, and therefore it should be possible for him to do the tapping with the boy.
When we had finished, we told the father that he should do the tapping and Havening as much as possible. Some days later we got a message from the interpreter: “I want to give you wonderful news. The father told me that he is using the tapping with the boy, and it is going extremely well! The father was super happy and the boy super calm and lovely.” The interpreter hugged us.

Along with their work with refugees, the Peaceful Heart Network has collaborated with local aid workers and groups in bringing TTT sessions and trainings to an estimated 250,000 people in 30 countries, addressing a wide range of post-disaster challenges. In Nepal, more than 900 tornado survivors received individual or group TTT sessions. In the most violent areas of Beni in the Democratic Republic of Congo, approximately 3,000 internally displaced persons, mainly youth and women, were served. Following Cyclone Idai in Zimbabwe, the Network reached approximately a hundred of the people whose homes had been swept away. In the world’s largest refugee camp, BidiBidi in Uganda, the Network has continuous engagement with refugees through a local pastor whose trainers have reached more than 2000 people since 2018. In Colombia, they have been training social workers in a group that supports victims of trafficking. Individuals and groups can connect with their services through https://peacefulheart.se/.

### California Fires

Kristin Miller, a clinical psychologist and a resident of Northern California, is a member of the Humanitarian Committee of the Association for Comprehensive Energy Psychology (ACEP). She has brought ACEP’s ‘‘Resources for Resilience’’^7^ and the TTT approach developed by the Peaceful Heart Network to those impacted by the devastating fires that have raged through her community year after year. She has found utility and efficiency in this “highly portable set of non-verbal, self-administered skills for managing stress and trauma.” She started by “showing up at every possible place where people were gathered and to listen to their stories and share skills.” Dr. Miller further reflected:

My stunning realization following wave after wave of outreach in my community was that no one had the skills to calm their survival system and to process the trauma. This was true whether I was working with the fire fighters, other first responders, hospital staff, county mental health providers, trained counselors, school staff, or the Red Cross shelters. But as these skills are becoming more embedded in our community, the road to recovery becomes more clear.
When the exhausted medical personnel, county workers, and fire fighters came off our blackened, treeless hills, we met them to process their post-event distress. Many had worked 70 and 80 days straight, trying to control the wildfires. Many had seen much death, some having to drive past dead bodies to get people out of towns that were on fire. Others had circled their trucks around people to keep the flames at bay as people were grappling for their lives.
All experienced feelings of powerlessness as the fires roared indiscriminately, dismantling all plans for an effective fire fight, as when the whole town of Paradise burned down in less than 2 h.
A fire supervisor came in completely unraveled with symptoms of traumatic distress. We did two long TTT sessions. I called him a few months later, and he said, “I am fine. That tapping was a great band aid!”
Employees of a utility company grieved the loss of colleagues while at the same time being blamed for causing the fire. In one incident within these dire circumstances, we used TTT in the hallways of a hotel and were able to tap through their trauma and clear their overwhelm before they proceeded to investigate the death of a crew member.
I came into a room of men in a Red Cross shelter just hours after they had escaped from the November 2018 Camp Fire (which caused 86 deaths) with only their lives. One man was in fight mode, angrily screaming into his cell phone. Another was rocking back and forth, trying to regulate his system. Another was checked out totally, frozen in a vacant stare. Another man seemed somewhat relaxed and open for engagement. He recounted gruesome collective stories about what the men in the room had experienced. I had him do some regulating breathing with me. Soon, one by one, each man joined in. We were then able to add some tapping. They all settled, and their nervous systems were regulated in about 20 min. Not long after, this team came out of their “cave” and began serving everyone else. While they had been as traumatized as all the others, they became a calming force within the shelter.
A more recent outreach was to medical staff at a hospital during the fifth wildfire of the 2021 season to visit our community. This one was during the COVID Delta surge. I stood with staff, tapping and breathing. They had suffered through days of 18-h shifts and multiple deaths in the hospital. People were surprised by how quickly they could regulate their nervous systems together, even in the midst of often fatal emergencies. The house supervisor walked me out in tears after a nurse, who had become a patient, had just succumbed. One of this supervisor’s heart-breaking responsibilities was to enforce the rule that no family is allowed on the COVID units. This triggered her own grief every time as she had not been able to be with her husband when he died. We sat and tapped and honored the grief together. A smile returned to her face.

Several additional members of the ACEP Humanitarian Committee have also been called to provide on-the-ground services following major wildfires. Others have been working with the trauma experienced by communities due to the pandemic. Some members have responded to mass shootings and other disasters. The Committee itself initiated the Veteran-to-Veteran Project in Nevada, training leaders in the veteran community to teach energy psychology self-help methods to other veterans to reduce the symptoms of combat-related trauma. Over a dozen groups now have “Peer Support Leaders” trained in TFT by the Veteran-to-Veteran Project, including the Veteran Transition Resource Center in Las Vegas, the Elizabeth Dole Foundation Hidden Heroes Program, a Veterans Administration regional hospital, and local chapters of the American Legion, the Veterans of Foreign War, and women vet groups.

The ACEP Humanitarian Committee also created “Resources for Resilience,” referred to above, a project that seeks to alleviate suffering by teaching self-help techniques to those experiencing the effects of violence, trauma, and natural disasters. The Resources for Resilience initiative includes a free emotional first aid training program and slides for community use, available from www.r4rtraining.support. The Committee has also, in collaboration with the Peaceful Heart Network, created a series of videos designed to help professionals who wish to become involved in humanitarian outreach projects. The Committee also partnered with the Peaceful Heart Network in training staff members from five different organizations, uniting medical doctors, psychologists, psychiatrists, and social workers in the use of TTT for working with Syrian refugees. This training was recorded on video with Arabic translation. The Committee has also partnered with Project LIGHT in bringing tapping to 48 facilitators who have worked with more than 400 youth and families in Rwanda.

### A Rwanda Orphanage

In 2006, Caroline Sakai, Suzanne Connolly, and Paul Oas conducted a study of TFT treatments with 50 Rwandan orphans who suffered with severe symptoms of PTSD (Sakai et al., 2010). The outcomes vastly exceed those of any existing peer-reviewed study of a single-session PTSD treatment in terms of speed, degree of effectiveness, and percentage of subjects who were helped. After a single session, 48 of the 50 children, many who had witnessed their parents being slaughtered during the genocide, were no longer in the PTSD range based on ratings on a standardized checklist completed by their care-givers prior to and following the treatment. Benefits were sustained on 1-year follow-up on the same measure. In the following account, Dr. Sakai described the experience of one of the study’s participants, a 15-year-old girl who was three at the time of the 1994 genocide:

She’d been hiding with her family and other villagers inside the local church. The church was stormed by men with machetes, who started a massacre. The girl’s father told her and other children to run and to not look back for any reason. She obeyed and was running as fast as she could, but then she heard her father “screaming like a crazy man.” She remembered what her father had said, but his screams were so compelling that she did turn back and, in horror, watched as a group of men with machetes murdered him.
A day didn’t pass in the ensuing 12 years without her experiencing flashbacks to that scene. Her sleep was plagued by nightmares tracing to the memory. In her treatment session, I asked her to bring the flashbacks to mind and to imitate me as I tapped on a selected set of acupuncture points while she told the story of the flashbacks. After a few minutes, her heart-wrenching sobbing and depressed affect suddenly transformed into smiles. When I asked her what happened, she reported having accessed fond memories. For the first time, she could remember her father and family playing together. She said that until then, she had no childhood memories from before the genocide.
We might have stopped there, but I instead directed her back to what happened in the church. The interpreter shot me a look, as if to ask, “Why are you bringing it back up again when she was doing fine?” But I was going for a complete treatment. The girl started crying again. She told of seeing other people being killed. She reflected that she was alive because of her father’s quick thinking, distracting the men’s attention while telling the children to run.
The girl cried again when she reexperienced the horrors she witnessed while hiding outside with another young child. The two of them were to be the only survivors from their entire village. Again, the tapping allowed her to have the memory without having to relive the terror of the experience.
After about 15 or 20 min addressing one scene after another, the girl smiled and began to talk about her family. Her mother didn’t allow the children to eat sweet fruits because they weren’t good for their teeth. But her father would sneak them home in his pockets and, when her mother wasn’t looking, he’d give them to the children. She was laughing wholeheartedly as she relayed this, and the translator and I were laughing with her.
We then went on to work through a number of additional scenes. Finally, when she was asked, “What comes up now as you remember what happened at the church,” she reflected, without tears, that she could still remember what happened, but that it was no longer vivid like it was still happening. It had now faded into the distance, like something from long ago. Then she started to talk about other fond memories. Her depressed countenance and posture were no longer evident.
Over the following days, she described how, for the first time, she had no flashbacks or nightmares and was able to sleep well. She looked cheerful and told me how elated she was about having happy memories about her family. Her test scores had gone from well above the PTSD cutoff to well below it after this single treatment session and remained there on the follow-up assessment a year later.

The powerful impact of the single sessions provided to each of the 50 adolescents surprised the investigators. Their plan for the study had included three treatment sessions, but an emergency in the country diverted half the clinical staff. Since the pre-tests had already been conducted, the investigators decided to proceed with the one session they were able to offer and hope for the best. The outcomes and the stability of the benefits on 1-year follow-up, according to my interviews with Dr. Sakai, exceeded their hopes.

The study was conducted in collaboration with the Trauma Relief Committee of the Thought Field Therapy Foundation, the first organization to systematically send disaster relief teams trained in acupoint tapping to trouble spots throughout the world. More than 20 of their past projects, including deployments to Rwanda, Haiti, Mexico, Uganda, the Democratic Republic of Congo, New Orleans, and Tanzania, are chronicled on http://www.tftfoundation.org/category/past-projects. The first clinical trial of TFT in treating people who had been repeatedly exposed to traumatic events (*N* = 31) showed a significant drop in all sub-groupings of PTSD symptoms (Folkes, 2002), and four studies investigating outcomes of the Foundation’s early projects demonstrated TFT’s speed and potency in post-disaster situations (Dunnewold, 2014). Dr. Sakai’s, 2014 book, *Overcoming Adversity: How Energy Tapping Transforms Your Life’s Worst Experiences – A Primer for Post-Traumatic Growth* is a poignant and insightful account of many of her experiences.

### Hurricane Katrina

A hurricane can wreak horrific devastation in minutes. On August 29, 2005, Hurricane Katrina hit New Orleans and the surrounding areas, causing more than 1,800 fatalities and $125 billion in damage. A team of twelve TFT practitioners from eight states was invited by three medical and social service organizations in New Orleans to provide treatment and training to their staffs 4 months following Hurricane Katrina. These medical and social service personnel were inevitably victims of the disaster as well as helpers, and the strategy taken was to make their treatment part of their training. A total of 161 participants received treatment and training at six different sites, with the largest number in an army tent at the Charity Hospital’s “MASH unit” in the New Orleans Convention Center. Written evaluations were obtained from 87 of the participants. Of these, 86 stated that they experienced positive changes and/or elimination of the problems they were experiencing at the time. Data compiled by Dr. Sakai (who had also done the work in Rwanda) on the 22 participants she treated showed that their presenting complaints included anger, anxiety, depression, eating to counter anxiety, frustration, guilt, survivor guilt, hurt, loss, loss of control, need for improved performance, overwhelm, panic, physical pain, resentment, sadness, shame, stress, traumatization, and worry. Each problem area was given the 0-to-10 SUD (Subjective Units of Distress) rating. Before treatment, the average (mean) score for the 51 problem areas described by the 22 clients was 8.14. After treatment, usually consisting of a single individual session of under 15 min (which followed a half-hour group orientation), it was down to 0.76, a remarkably large decrease.

In addition to the TFT team, EFT practitioners also worked in the immediate aftermath of the hurricane and later with those who had been displaced. Sophia Cayer, who is highly experienced with tapping, described to me her experience with a woman who had not only been traumatized by the hurricane, but also by her subsequent time in a shelter after her home had been destroyed. A month after Katrina, she was so depressed that she was unable to function, spending much of her time crying uncontrollably. Cayer continued:

When I sat down with her, she had one hand over her face, sobbing and unable to speak. I gently asked for permission to take her hand and see if I could help her relax. She agreed, and I began gently tapping on the energy points on her hand. Within a few moments, her tears began to subside. She was still unable to voice her experience, so I just kept tapping and talking with her. I used a specific EFT technique which offers relief without the person having to verbally describe the event. Among other issues, she was haunted by the screams and sounds of gunshots during the nights she spent in the shelter. While she was still, for the most part, unable to speak, I continued working with her, with her tears coming and going. After several minutes, her head was held high and she was able to speak. Then she smiled. Later that evening, I saw her at a gathering for survivors. Her friends, who had initially put me together with her, seemed amazed, reporting that she was her cheerful self again. I will always remember her smiles and hugs of gratitude.

Cayer reflected that with tapping, “even if it is only a single session, it doesn’t leave the person stranded. It is not a matter of just soothing them and then letting them go. They are given powerful tools they can regularly use as they move through the crisis and beyond.”

### Combat Veterans

After learning of the first RCT demonstrating the effectiveness of tapping protocols in the treatment of 49 combat veterans (Church et al., 2013), I contacted the study’s principal investigator and asked whether I could interview some of the therapists involved. I wanted to get a sense about the *experiences* summarized in the statistics. One of the therapists, Ingrid Dinter, described to me her work with Keith, an infantry soldier who had served in the Mekong Delta during the Vietnam War. He reported that in his initial therapy session with her that he had seen “many casualties on both sides.” More than three decades later, he was still tormented with nightmares and repeated flashbacks: “Sometimes I think I see Viet Cong soldiers behind bushes and trees.” His severe insomnia, complicated by the nightmares, made him fatigued and unable to function during the day. He’d been diagnosed with PTSD and reported that his group and individual therapy through the Department of Veterans Affairs (VA) hadn’t helped with his symptoms.

Keith received 6 h-long sessions with Ingrid, during which she had him tap on acupoints while he focused on traumatic war memories and other psychological stressors. In their first session, he reported that since the war’s conclusion, he’d rarely gotten more than 1 to 2 h of sleep at a stretch and averaged about two nightmares each night. By the end of the six sessions, he was getting 7 to 8 h of uninterrupted sleep and was having no nightmares. He said that other symptoms, such as intrusive memories, startle reactions, and overwhelming obsessive guilt had abated as well. A 6-month follow-up interview and further testing showed that the improvements held.

At this point, more than 21,000 veterans have received free or low-cost tapping sessions from the Veterans Stress Solution^8^. A 10-min clip containing brief excerpts of interviews with four combat veterans before and after energy psychology treatments, along with snippets from the treatments they received, can be found at www.vetcases.com.

### Earthquakes and Floods

A 2010 earthquake in Haiti caused more than 200,000 deaths and eight billion dollars in damage. Seventy-seven of the survivors were assessed for PTSD on a standardized symptom inventory. Forty-eight scored in the clinical range. After two days of instruction in EFT, none of the participants showed a score in the clinical range on post-test (*p* < 0.001). Posttest symptom and symptom severity scores decreased by an average of 72%, ranging between a 21% reduction and a 100% reduction (Gurret et al., 2012). Another team went to Haiti 6 months after the earthquake to provide training in TFT. In addition to reporting on the effectiveness of the tapping treatment, they discuss challenges, such as limited healthcare resources and poor infrastructure, that had to be overcome for the program’s success (Robson and Robson, 2012).

An earthquake in Indonesia in 2006 killed more than six thousand people and destroyed 60,000 homes. The Tapas Acupressure Technique^®^ (TAT), an energy psychology approach in which acupoints are held (rather than tapped) during the verbal component, was taught to local relief workers, leading to some six thousand adults and children receiving the treatment in individual and group settings. The Mexican Association for Crisis Therapy has used TAT in training hundreds of frontline service personnel working with floods and other natural disasters in Mexico, Nicaragua, Venezuela, and Columbia. Ignacio Jarero, the Association’s president, stated on the TAT website, ‘‘Children and adults reported significant reductions in SUD scores at the completion of the protocol.... TAT is our favorite technique to reduce distress because it is easy to teach and apply’’ (^9^ retrieved February 10, 2022).

## Efficient Delivery of Energy Psychology Services

In the wake of a disaster, community and emergency response resources are often overwhelmed. When hundreds or thousands or tens of thousands of people have been affected, the need for delivery systems that are more efficient than individual counseling emerges with unyielding urgency.

A meta-analysis of 10 therapies treating PTSD in adults found that the one which used an acupoint tapping protocol was more cost-effective than trauma-focused CBT, selective serotonin reuptake inhibitors (SSRIs), and six of the other seven therapies that were evaluated (Mavranezouli et al., 2020b). A reason for this cost-effectiveness is that energy psychology treatments tend to work so rapidly in reducing trauma-induced limbic system hyperarousal. They are also highly flexible in that they can be delivered as one-on-one therapy provided by professionals or lay counselors, in classes and workshops, online as tele-therapy, and/or taught as a self-help technique.

Four approaches that have emerged for providing energy psychology services more efficiently involve the use of large groups, lay counselors, digital delivery, and training local professionals and institutions to field the long-lasting effects of a major catastrophe. Each is discussed in this section.

### Large Groups

The energy psychology approaches that are in widest use for disaster relief (EFT, TAT, TFT, TTT) have all been applied in group as well as individual formats. Simple energy techniques for self-soothing can quickly reduce the symptoms of acute stress and be efficiently taught in group settings. Because it is not necessary to verbalize their traumas in order to experience benefits from basic energy psychology procedures, participants are able to obtain immediate relief without having to reveal to other group members specific memories or emotions.

A group approach that takes participants beyond the first tier of energy psychology interventions in the aftermath of a disaster (immediate relief/stabilization), though still anonymously for most group members, has also been developed. It involves having each person bring to mind a troubling memory or emotion and giving it the 0-to-10 SUD rating. A volunteer from within the group is then selected for a tapping session, led by a trained practitioner while the group watches. At the same time, group members are instructed to shift their focus from their own issues to the volunteer’s psychological distress. They self-apply the procedures being used with the volunteer as if the volunteer’s issue were their own issue. This causes the volunteer to feel the focused support of the group while the group members are experiencing attunement and compassion for the volunteer. They are also witnessing, practicing, and further internalizing the tapping procedure. The approach also has an unexpected effect. When the group members go back to re-assess their own initial situation by giving it a second SUD rating, most of them find that the amount of emotional intensity or distress has diminished.

Called “borrowing benefits,” this phenomenon is widely reported by tapping practitioners and has been investigated. A within-subjects design was used with 102 participants who attended either of two 3-day Borrowing Benefits workshops conducted by EFT originator, Gary Craig (Rowe, 2005). The participants were given a well-established, standardized symptom checklist 1 month prior to the workshop, immediately prior, immediately after, 1 month after, and 6 months after the workshop. No significant difference was found in the mean test scores 1 month prior to vs. immediately prior to the workshop. Following the workshop, however, a highly significant decrease (*p* < 0.0005) was found on the checklist’s global measure of psychological distress as well as all nine subscales. These improvements held at the 6-month follow-up. The majority of the participants did not experience individual treatments during the workshops. While the mechanisms for such positive “contagion” have not been established, practitioners are consistently describing similar findings (Church and House, 2018). The value of utilizing such an effect following a mass disaster—where survivors have gone through parallel experiences—is obvious.

For example, during Dr. Leyden’s work in Rwanda, the organization that arranged which villages and which groups she would meet with had allotted six days for her to work with a hundred orphans who were also heads of their households. They ranged in age from 19 to 25. At the time of the genocide, they were only 5 to 11 years old, yet they were left to care for two to six other orphans with no visible means of support for food, rent, education, or daily living expenses. With close to 100,000 orphans from the war and hundreds of thousands more children being orphaned as a result of their parents dying of HIV/AIDs, the Rwandan government did not have the resources or capacity to care for these orphans.

Since individual counseling with 100 people over a six-day period wasn’t going to go very far, Leyden decided to meet with the entire group on each of the six days. She was still able to have one-on-one sessions, but she could amplify their impact by conducting them in front of the large group. Using the borrowing benefits model, everyone in the group would, before each one-on-one session, tune into their own area of greatest concern, give it a SUD rating, and then bring their attention to tapping along with the person working with Dr. Leyden in the front of the room, using the wording that person used.

The first individual session was with a young man, about 20 years of age, who had been caring for three other orphans since the time of the genocide. He was still struggling with intrusive memories about the genocide 14 years earlier. He rated his distress as being “above 10” on the 0-to-10 SUD scale. He, Dr. Leyden, and the entire group tapped on these and similar statements, one at a time:

• My mind does not feel safe.
• My memories will never go away.
• I will be terrorized by these memories for the rest of my life.
• I’m afraid to sleep because of these memories.

After 25 min of tapping, he reported that he was at a two on the SUD scale. He commented, “my mind feels safe now. I know I am safe right now. I’m looking forward to sleeping tonight.” Meanwhile, Dr. Leyden reported sensing a palpable shift in the room. There was a quiet sense of peacefulness that wasn’t there before the session. As she checked in with the rest of the group, all one hundred participants reported being at a two or below on their original issue. Specifically, one person after another reported feeling safer in his or her body than they could ever remember having experienced.

As the week progressed, the young man reported that his sense of calm persisted at home each evening. He was able to sleep peacefully. Others who had one-on-one sessions addressed additional themes, including these, as the group tapped along with them:

• Helplessness of being an orphan.
• Hopelessness of not having a bright future to look forward to.
• Healing for the wound of rape.
• Terror about the return of intrusive memories of their parents and families being murdered in front of them.
• Pain of often going hungry.
• Anxiety of not being able to provide basic needs for the younger children they were caring for.

During the sessions, the participants reported how the new sense of safety helped them cope better with the challenges they faced on a daily basis. They also taught their “families” to tap at home, which made them feel more in control of their situation and better able to help other family members when they were feeling stressed or traumatized. Dr. Leyden commented that it was for her, “another reminder of what I’ve seen hundreds of times: the power of the tapping to re-regulate the physiological dysfunction that occurs when a person is traumatized.”

### Lay Counselors

Since so many more volunteers can be made available for disaster relief efforts than trained mental health professionals, efforts to quickly and efficiently train laypeople to provide safe and effective emotional support services have been carried out and appear promising. A study published in the *Bulletin of the World Health Organization* reviewed 20 clinical trials involving 5612 participants. It investigated outcomes following a variety of treatments provided by lay counselors, with a focus on PTSD, depression, anxiety, and alcohol use. The review concluded that the “use of professionally trained lay counselors to provide mental health interventions in low- and middle-income countries was associated with significant improvements in mental health symptoms across a range of settings” (Connolly et al., 2021, p. 572).

An analysis of the practitioners in a study of EFT in the treatment of PTSD with combat veterans was conducted by Stein and Brooks (2011). Of the 59 volunteer practitioners, 26 were licensed mental health counselors (LMPs) and 33 were “lay coaches.” Based on the selection criteria of the original study, all of the veterans had scored above the PTSD cutoff on a PTSD inventory that had been standardized for veterans. After six sessions, 90% of the veterans treated by the LMPs no longer scored in the PTSD range as contrasted with 83% who were treated by the lay coaches. This trend for better outcomes with the LMPs did not reach statistical significance, and the authors concluded that EFT provided by lay coaches was “an effective strategy to address PTSD in this population” (p. 11).

Two clinical trials of TFT treatments following the genocide in Rwanda (Connolly and Sakai, 2011; Connolly et al., 2013) and the report of the use of TFT after the 2010 earthquake in Haiti (Robson and Robson, 2012) were notable not only for their effectiveness. They each also used lay counselors to provide the services.

In the 2011 Connolly and Sakai study of 145 survivors of the Rwanda genocide, the 28 community leaders who administered the treatments each received two full days of training in TFT, including hands-on practice and hands-on supervision while providing the treatments. The participants were randomly assigned to a single-session treatment group or a no-treatment condition. All reported PTSD symptoms that had persisted for more than a decade after the genocide (which was one of the selection criteria). These symptoms were significantly reduced (*p* < 0.001) for the treatment group but not for the controls. Gains were maintained at 2-year follow-up. The study’s authors concluded:

While it is desirable to utilize experienced mental health professionals in treating severe PTSD, the possibility of enlisting community leaders to treat fellow community members employing an efficacious, non-narrative therapeutic modality that does not require years of clinical training vastly enhances the potential mental health care resources in a community devastated by large-scale trauma (p. 171).

The Connolly et al. (2013) study was a partial replication of the 2011 investigation, again using a single-session design with lay counselors who had been provided with two days of TFT training for treating trauma symptoms, working with a different population of Rwanda genocide survivors. As with the earlier study, self-reported symptom reduction was highly significant for the treatment group.

Having reached some 250,000 individuals who have experienced disasters in more than 30 countries, the Peaceful Heart Network probably has the most experience in utilizing large groups and lay counselors in bringing tapping to post-disaster areas. They explained to me how their reach multiplies so quickly: “One of our trainings in an African village for self-applying TTT was attended by 40 people. We asked each participant to teach 4 others. We also requested that they ask each of the people they teach to teach four more. Within 2 months, TTT had spread to several thousand people in the area”. Hamne and Sandström’s (2021) book, *Trauma Tapping Technique: A Tool for PTSD, Stress Relief, and Emotional Trauma Recovery* (2021), outlines their approach.

### Digital Delivery

The COVID-19 pandemic caused many psychotherapists to innovate ways for delivering their services online. Growing evidence shows that telehealth delivery of mental health services can be effective. A recent meta-analysis found that in some circumstances online cognitive behavioral therapy was more effective than face-to-face sessions (Luo et al., 2020). Acupoint tapping protocols are particularly amenable to online treatments, and digital delivery has been used with the approach for well over a decade (Church, 2012). Church listed numerous advantages of online treatment and self-help resources, such as greater reach, user access to 24/7 support, anonymity, and lower costs. He also discussed potential disadvantages, such as the possibility of abreactions or retraumatization with no immediate hands-on support, inappropriate self-treatment for severe mental health diagnoses like schizophrenia or bipolar disorder, and a lack of quality control or unclear accountability for the developers and providers of online services.

The first systematic study of digital energy psychology treatments showed that an 8-week online EFT program significantly reduced pain, anxiety, and depression while improving vitality, social function, and mental health measures in 26 women diagnosed with fibromyalgia (Brattberg, 2008). The majority of the 21,000-plus tapping sessions provided by the Veterans Stress Solution (see text footnote 8) have been online. The ACEP Humanitarian Committee’s “Resources for Resilience” program posts a variety of online tools that can help “alleviate suffering by teaching self-help techniques to those experiencing the effects of violence, trauma, and natural disasters” (see text footnote 7). A cell phone app that guides users in applying acupoint tapping protocols for anxiety and stress was investigated in a large-scale study including 270,461 app users and found highly significant (*p* < 0.001) symptom reduction (Church et al., 2020).

An online format can not only make services more accessible, it can also facilitate large group sessions. The ACEP Humanitarian Committee members reported in an informal survey the frequent use of group Zoom sessions or telehealth classes. In an RCT investigating outcomes of EFT treatments on stress, anxiety, and burnout levels during the pandemic, 72 nurses were randomized into a tapping group or a no-treatment control group (Dincer and Inangil, 2021). Based on a single guided online group EFT session, reductions in stress level, anxiety, and burnout indicators reached high levels of statistical significance (*p* < 0.001). The control group showed no statistically significant changes on these measures.

### Training Local Professionals and Institutions

A single major catastrophe can change a community forever. While disaster relief teams coming in from other areas can bring indispensable resources, their time in the community is relatively brief. Energy psychology disaster relief teams from Newtown to Parkland to Rwanda to Aboriginal communities in Australia have been evolving ways for training and empowering the local leaders and disaster survivors for enhancing the community’s long-term resiliency. Before their departure, relief teams are increasingly providing training in acupoint tapping protocols to local mental health professionals and agencies who will be serving the long-term needs of their communities. For example, following the high school shooting in Parkland, Florida, which is part of Broward County, a community of 2.2 million that was deeply affected, trained professionals are now providing EFT services in 20 agencies in the county. This was made possible because of recognition by government agencies as well as private foundations—including the local Children’s Services Council and the New York Life Insurance Foundation—of the need to scale and replicate tapping therapies so they would be widely available.

### Trends in Service Delivery

Based on my interviews with practitioners who have incorporated the stimulation of acupoints into disaster relief work, these four trends have been appearing for increasing the impact of energy psychology interventions. Supporting the first two trends (large group applications and the use of carefully selected community members so they can be taught the method and then teach it to other survivors with the response team’s supervision) has been a tendency to simplify tapping protocols. This allows tapping protocols to be readily and widely applied for immediate relief and restabilization. A primary way of accomplishing this has been to focus the tapping on the *felt sense* (after Gendlin, 1982) of traumatic memories or other disturbing emotions rather than relying on verbal descriptions or formulistic phrasings. The third trend, utilizing digital technology, is appearing both for making the best instruction for coping with a disaster widely available and also for conducting personally tailored treatment sessions remotely with individuals and groups. All three of these developments may be utilized in the fourth trend, which is to empower the local community to address ongoing mental health challenges and to develop greater resilience from having lived through the tragedy.

## Training

Training and certification in the various forms of energy psychology are available from numerous organizations. Among the largest and most widely respected include the Association for Comprehensive Energy Psychology^10^, EFT Universe^11^, Thought Field Therapy^®12^, EFT International^13^, and Evidence Based EFT Training^14^. In addition to the basic skills necessary for addressing psychological issues utilizing acupoint tapping protocols, training for serving as a disaster responder is widely available from organizations such as the Red Cross^15^, the Green Cross Academy of Traumatology^16^, and the International Trauma Training Institute^17^. A list of other in-person and online courses can be found at https://www.apa.org/practice/programs/dmhi/dmh-training/disaster-mental-health-training. More than a dozen books are available for examining the issues any disaster responder needs to understand. Three of my favorites are the already-mentioned *Interventions Following Mass Violence and Disasters* (Ritchie et al., 2007), *The Body Keeps the Score* (van der Kolk, 2014), and *The Worst Is Over: What to Say When Every Moment Counts* (Acosta and Prager, 2014).

## Future Research

Additional systematic research is called for as growing numbers of disaster relief teams are utilizing energy psychology protocols. Among the topics needing further investigation: (a) Are energy psychology interventions in the immediate aftermath of a disaster more effective than other modalities in preventing post-traumatic symptoms long-term? (b) Which energy psychology strategies are most effective during each phase of post-disaster recovery? (c) What adjustments need to be made based on the type of disaster? (d) When is it best to utilize or not utilize strong reliance on the verbal components of energy psychology protocols? and (e) How can efficient delivery approaches such as the use of large groups and digital technology be fashioned to enhance rather than dilute the effects of the method?

## Conclusion

The escalating incidence of natural and human-made disasters has resulted in a critical shortage of mental health resources for addressing the emotional consequences and compromised functioning of millions of people who have lived through catastrophic events worldwide. Applications of energy psychology interventions following disasters in more than 30 countries have been promising, and efficient means of delivery include large groups, lay counselors, digital technology, and community empowerment. Reports from those providing these services as well as a limited number of empirical studies appear compelling, with the technique’s ability to quickly ease the neurological aftermath of trauma standing out as a major advantage.

## History and Permissions

This article is a thorough revision and update, adapted from the author’s 2008 “Energy Psychology in Disaster Relief,” published in *Traumatology*, 14(1), 124–137. Copyright © 2008 by the American Psychological Association. Selected passages reproduced with permission.

## Author Contributions

The author conducted each stage in the development of this manuscript.

## Conflict of Interest

DF conducts trainings, provides clinical services, and has written books related to the approach examined in this manuscript.

## Publisher’s Note

All claims expressed in this article are solely those of the authors and do not necessarily represent those of their affiliated organizations, or those of the publisher, the editors and the reviewers. Any product that may be evaluated in this article, or claim that may be made by its manufacturer, is not guaranteed or endorsed by the publisher.

## Footnotes

1
https://cmbm.org/

2
https://www.energypsych.org/researchdb8c71b7

3
https://capacitar.org/

4 https://www.thetappingsolution.com/, retrieved February 10, 2022.

5 https://www.createglobalhealing.org/, retrieved February 10, 2022.

6 https://peacefulheart.se/, retrieved February 10, 2022.

7 https://www.r4r.support, retrieved February 10, 2022.

8
https://www.stresssolution.org/

9
https://tatlife.com/disaster-relief/

10
https://www.energypsych.org/

11
https://eftuniverse.com/

12
https://tfttapping.com/

13
https://eftinternational.org/

14
https://evidencebasedeft.com/

15
https://www.redcross.org/take-a-class/disaster-training

16
https://greencross.org/

17
https://traumaonline.net/certified-clinical-trauma-professional-cctp/
